# Clarity, conviction and coherence supports buy-in to positive youth sexual health services: focused results from a realist evaluation

**DOI:** 10.1186/s12913-019-4298-4

**Published:** 2019-07-19

**Authors:** Katie Shearn, Hilary Piercy, Peter Allmark, Julia Hirst

**Affiliations:** 1 Department of Nursing and Midwifery, Faculty of Health and Wellbeing, Sheffield Hallam University, 32 Collegiate Crescent, Sheffield, S10 2BA England; 2 Department of Psychology, Sociology & Politics, Sheffield Hallam University, HC 2.03a, Heart of the Campus Building, Collegiate Crescent Campus, Sheffield, S10 2BQ England

**Keywords:** Realist, Programme theory, Sexual health, Young people, Organisational change

## Abstract

**Background:**

There is a call for sexual health services to support young people achieve sexual wellbeing in addition to treating or preventing sexual ill-health. Progress towards realising this ambition is limited. This study aimed to contribute theory and evidence explaining key processes to support local delivery of positive youth sexual health services.

**Methods:**

A realist evaluation was conducted, comprising four research cycles, with a total of 161 data sources, primarily from the UK. Theory was refined iteratively using existing substantive theories, secondary and primary research data (including interviews, documentary analysis, feedback workshops and a literature search of secondary case studies). A novel explanatory framework for articulating the theories was utilised.

**Results:**

The results focused on local level buy-in to positive services. Positive services were initiated when influential teams had *clarity* that positive services should acknowledge youth sexuality, support young people’s holistic sexual wellbeing and involve users in design and delivery of services, and *conviction* that this was the best or right way to proceed. How positive services were operationalised differed according to whether the emphasis was placed on meeting service objectives or supporting young people to flourish. Teams were able to effect change in local services by improving *coherence* between a positive approach and existing processes and practices. For example, that a) users were involved in decision making, b) multi-disciplinary professional working was genuinely integrated, and c) evidence of positive services’ impact was gathered from a breadth of sources. New services were fragile. Progress was frequently stymied due to a lack of shared understanding and limited compatibility between characteristics of a positive approach and the wider cultural and structural systems including medical hegemony and narrow accountability frameworks. These challenges were exacerbated by funding cuts.

**Conclusions:**

This study offers clarity on how positive youth sexual health services may be defined. It also articulates theory explaining how dissonance, at various levels, between positive models of sexual health service delivery and established cultural and structural systems may restrict their successful inception. Future policy and practice initiatives should be theoretically informed and address barriers at societal, organisational and interpersonal levels to stimulate change.

**Electronic supplementary material:**

The online version of this article (10.1186/s12913-019-4298-4) contains supplementary material, which is available to authorized users.

## Background

### The call for positive, comprehensive youth sexual health services

There is a widespread call for positive, comprehensive youth sexual health services (hereafter referred to as ‘positive services’) which support young people to achieve sexual wellbeing as opposed to just preventing ill health. This call is apparent in international [1–3], United States [4], English [5] and other UK national policies [6–8]. It is endorsed by academics from a variety of disciplines [9–13], policy advisors and advocates for young people [14–16]. Furthermore, young people themselves demand services which are ‘sex positive’ [17–21] (as opposed to ‘sex negative approaches’ [22]), which encapsulate notions of diversity, empowerment and choice in relation to sexuality [23].

A range of scholars have produced frameworks of principles to underpin policy, practice and research to support the attainment of sexual wellbeing [23–25]. Some examples of positive services in practice challenge the traditional models of disease control and prevention. Commonly these examples acknowledge young people’s sexuality and a broader conceptualisation of sexual health [26–28], frame young people’s sexual health in the context of sexual rights [29], positive health [11] and address wider determinants of health relating to individual, relational, community and societal ‘levels’ of social structure in programming [24]. They also stress that users influence the design of services. These cases and frameworks do not, however, detail *how* existing services might be transformed to deliver against these principles. That is, theory and evidence explaining how to operationalise these principles are lacking.

### The need for theory and evidence to support service transformation

Some academics [11, 30] and policy bodies [1, 31] describe a general lack of progress towards positive services. The World Health Organization [1] recognised that both theoretical and empirical work is needed to support this transformation. The aim of this study was therefore to elicit and/or develop programme theory, (which we define as the explanation of how an intervention is intended to lead to a goal [32–36]), which could support the widespread development of positive services and explain the current limited progress.

The overarching research question was ‘what works to deliver positive youth sexual health services, when, under what circumstances and why?’ This article concerns theory and evidence relating to one aspect of implementation, namely, ‘buy-in’ to positive services; that is, that individuals within the local sexual health services system, (within which we include frontline workers, managers, and commissioners) distinguish, value and are prepared to invest energy in delivering them. We acknowledge that this provides only a partial explanation in response to the overall research question. Recommendations on developing programme theories acknowledge that they can only ever be partial and fallible [37, 38]. Furthermore, we argue that detailed and nuanced reflection of key stages in implementation is essential to realise ambitions of accumulating knowledge about key mechanisms [37, 39].

## Methods

### Realist explanatory framework

A realist approach was utilised to develop and test programme theories; this approach is sensitive to complex systems, such as sexual health services, with multiple actors, processes, practices and emergent properties [32, 40]. The distinctive characteristics of realist philosophy that support this are rehearsed elsewhere [32, 41, 42]. We wish to distinguish, however, between the explanatory framework utilised in this study and that which uses Context, Mechanism and Outcome configurations (CMOc) and is generally accepted to be the hallmark of a Realist Evaluation [38] (but not without contention [39, 43]). One key difference is in the definition and usage of the term ‘mechanism’. Typically the definition attributed to mechanisms in the CMOc relates to the reasoning of actors in response to a resource [38, 43]. Westhorp [44] has recently elaborated upon this and similarly this study recognises that causal forces emanate from structural (e.g. processes, roles, practices, resources), cultural (e.g. ideas, norms) and agential (e.g. beliefs, skills, knowledge, reasoning) domains [45–48]. In other words mechanisms exist within cultural ideas and social structures, not just in the reasoning of agents.

Accordingly, theories developed within this study will utilise the following explanatory framework referring to:

- prior (and relatively enduring) cultural, structural and agential conditions which have effects, through conditioning, on individuals’ actions.
- habitus and/or knowledge, skills and internal conversations of actors which lead to actions.
- emergence of transformed agential, structural or cultural states, or alternatively reproduction of ‘prior’ conditions.

We turn now to outline the methods used to develop and refine programme theory relating to individual buy-in to positive services.

### Four cyclical research cycles

Theory was developed for this evaluation over four iterative research cycles between December 2015 and July 2016. Full detail of the methods are available elsewhere [32, 49, 50]. Ethics approval was granted by Sheffield Hallam Research Ethics Committee: proposal number HWB-HSC 35. All participants gave informed consent to participate in the study. Research governance was granted by the local NHS departments.

**Fig. 1:**
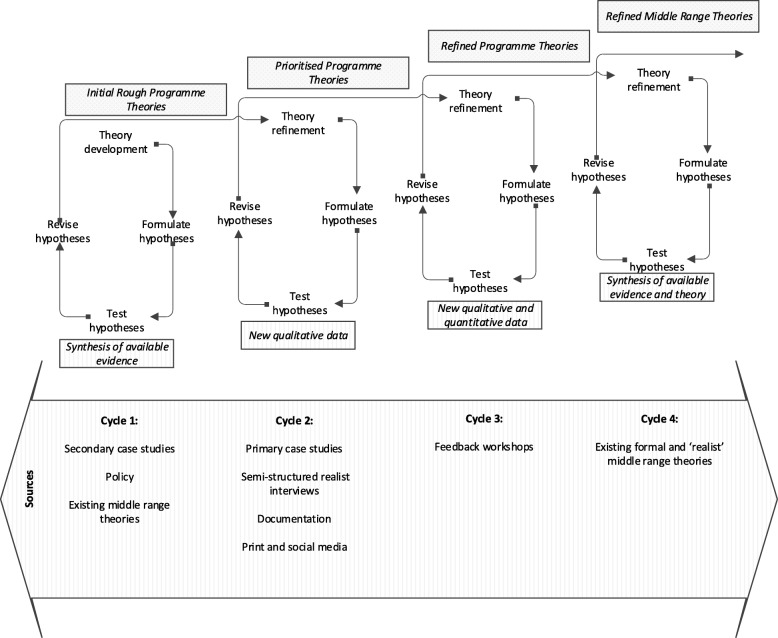
Overview of methods and sources

Figure 1 illustrates these cycles alongside key sources of data and existing theory:

### Cycle 1: establishing initial rough programme theories (IRPTs)

Secondary case studies of positive sexual health services were identified through a search of four academic databases (Scopus, MEDLINE, CINAHL Complete and Psychinfo) reference and citation searches (full details of the search strategy can be found in the published abstract [49], thesis [49] and Additional file 1) and hand searches of national and international policy archives. A working definition of positive youth sexual health services was established through a synthesis of this data.

Initial rough programme theories (IRPTs) were then developed to provide a nascent explanation of what works to deliver positive sexual health services. A scaffold of existing middle-range theories (MRT), the Morphogenetic Approach [45], Normalisation Process Theory (NPT)^1^ [51, 52] and COM-B from Michie’s Behaviour Change Wheel [53], was built to provide a broad conceptual framework within which to situate theories specific to the research aim (full details of this method can be found in our sister article [32]). Concepts from these theories were mapped to data from the literature. Initial propositions to explain how and why positive services might be implemented were developed using abductive and retroductive inferences. The IRPTs relevant to this aspect of the evaluation are provided in Additional file 2.

### Cycles two and three: refining programme theories

Cycle two was designed to gather more data to support the exploration of culture, structures and agency operating in youth sexual health services [54–57]. Primary case studies of current English NHS sexual health services, which catered for young people and described their service as a positive (or equivalent) model in marketing collateral, were purposively recruited. Data was collected using 24 semi-structured realist interviews [58] with commissioners, managers and frontline practitioners, recruited via an opt-in process (see Additional file 3 for full breakdown of numbers and categories, for details of interview guides see [50]). In addition, data was collected from six network meetings, service specifications, evaluations, print and social media and academic outputs.

Cycle three was a further round of data collection with each of the primary case studies, involving sexual health managers, nurses, consultants, administrators and social workers, to explore theories concerning buy-in. Feedback workshops were organised with each site. The emerging results and initial theories were translated into a brief presentation delivered by the first author. Feedback was gathered via booklets where participants could annotate the theories and identify their agreement or otherwise with them and note taking in the discussions. Forty seven of sixty three participants returned booklets across three sites which were given pseudonyms: *‘Ponston’, ‘Rissfield’* and *‘Stadford’.* All participants took part in discussions (see Additional file 3 for full breakdown).

Data, from both cycles, was analysed sequentially against a coding framework based on the IRPTs in NVivo version 10. Where the data did not fit the IRPT directly, but was relevant, the IRPT was tentatively elaborated (by adding more detail to this specific aspect of the theory) or new codes were added [59]. Over the cycles, confirmatory data, suggesting that the causal mechanism postulated could be considered more likely, was seen as strengthening the theories; its opposite, dis-confirmatory data, presented the opportunity to develop alternate explanations [60, 61]. In this way, IRPTs were prioritised and enhanced to produce refined programme theories (RPT).

### Cycle four: refining middle range theories

The concepts from the RPTs were mapped back to the original MRTs that informed the IRPTs. Where concepts had been added or elaborated (and were not described in the original MRTs), additional MRTs were sought to further enhance the explanatory power of the RPTs.

## Results

There were 161 data sources for this study: policy, literature and data from three secondary case studies and documentation and interviews from three primary case studies where local services had attempted to implement positive youth sexual health services. The full breakdown of sources is provided in Additional file 3.

**Fig. 2:**
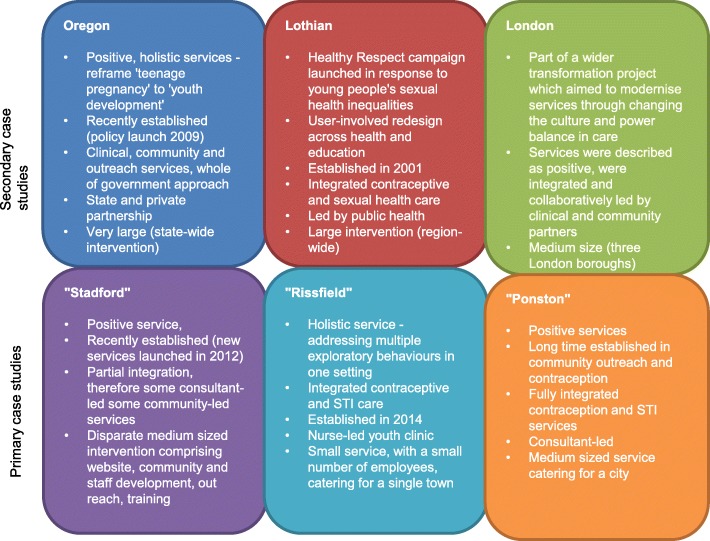
Pen portraits of services that had attempted to implement positive services

Cases were at different points in their proposed trajectory towards, or away from positive services. This enabled the project to consider the development of a positive approach over time as well as under different circumstances. Characteristics of the six cases are described in Fig. 2.

We will refer to these case studies to illustrate the theory development below.

The following results section is divided into two parts. The first two theory themes, *clarity* and *conviction* are combined and relate to theories of the internal conversations of social actors (conditioned by structure and culture). The second section relates to *coherence,* in this case the emergence, or otherwise, of changed structural and cultural states which may facilitate a positive approach to youth sexual health services. Each section will outline the data and then the RPTs.

### Clarity and conviction

#### Empirical evidence relating to clarity and conviction

The IRPTs relating to clarity suggested that a positive model should be distinguished from existing models of care. The data showed that such differentiation was important for local buy-in to positive services, but that varying interpretations of positive services were apparent, contingent on the context in which social actors were operating. Three interpretations are described briefly below.

1. *Positive services are ‘a quality marker’*

Some participants suggested a “positive” service was a quality marker overlaying a medical model*.* They acknowledged young people’s sexuality and recognised the necessity of services being welcoming and non-judgemental. For instance, they referred to the *You’re Welcome* criteria [5, 62] which clarified the concept and competencies of being ‘young-person-friendly’ [63]. Some clinical staff suggested that services were transactional - providing the young person with what they ask for, such as a pregnancy or STI test. If sexual history-taking revealed other needs, participants suggested they would try to signpost to other services or offer a brief intervention but as this was not the primary aim of the consultation it may not be followed up due to time pressures or lack of connectivity with other agencies. These participants considered other features of sexual health support, such as skill building to be more relevant to education or social work than to their own practice, and could risk de-skilling trained staff. For example, when describing possible sexual health work one clinician suggested that,

*'…promotion of sexual health is encouraging people to use condoms …contraception, giving support for …psychological or mental health issues. You need to move it upstream really so that they don't end up coming into the Health Service' (Consultant, 'Ponston').*

This division in roles is arguably at odds with services/practitioners being responsive to the diverse and holistic needs of young people that might be indicated by a positive approach. As such, these individuals’ interpretation of positive services did not differentiate from a medical model of sexual health care. This interpretation was largely, although not exclusively, held by GUM staff working in hospital settings who, in this sample, had less opportunity to develop relationships with the young people in their care and felt more restricted in the format of the consultations they could provide.

2) *Positive services are ‘a strategy to reduce sexual ill-health’*

Some participants perceived a positive approach to youth sexual health as embracing young people’s sexuality, broadening activities and involving users in design, as a means to encourage healthy behaviour. They felt that activities to build relationships and skills and work to promote gender equality would reduce incidence of infections, unwanted conceptions, child sexual exploitation and peer-on-peer violence. For example, this was supported in ‘Rissfield’ when practitioners were encouraged to conduct full, extended, unscripted and holistic consultations to cover all the aspects of sexuality and wider determinants that were important to young people.

This interpretation was widely held, most notably by those accountable for progress made on key sexual health indicators in England, namely, the under-18 conception and chlamydia positivity rates [5, 64]. Many had learnt about positive services through specific training, trusted evidence sources or peer networking. Their subsequent conviction was based on a perceived efficacy of such an approach meeting their organisational and role objectives, built on logic or evidence presented through these sources.

3) *Positive services are a means to promote choice and capabilities*

The third interpretation was that positive services should provide a means to promoting young peoples’ choices and capabilities. This interpretation, particularly apparent in Oregon, but also held by some in each of the case study sites, challenged the dominant power structures and cultural norms concerning young people’s position in society.

*'[it's a] coaching culture…that’s about facilitative…self-determination…self-actualisation. It's not about telling them what to do; it's about them realising what's best for them, what's possible.' (past community manager, 'Ponston')*

Participants with this view, especially those in community outreach, suggested a positive service supports young people to achieve their own personal brand of sexual wellbeing, recognises young people’s sexuality as integral to their health and wellbeing and a central part of their development and transition to adulthood.

In Stadford, commissioners and managers spoke of ‘changing their language’ away from a risk-based discourse. Here, positive services were interpreted as a need to provide universal, wide-ranging, low level sexual health support and advice for all whether or not they fall into ‘high risk’ categories. In addition, aspects of pleasure were foregrounded by providing interactive web pages for young people to explore ‘pleasure zones’ of the male and female body. This included pleasurable sexual sensations which could be attained without intercourse.

Frontline practitioners, notably those who had experience of community outreach, had come to an understanding of positive approaches through their experience working with young people.

*"…from the views that I may have picked up from young people over my time in this job and previous roles…" (Health Promotion Manager, 'Ponston')*

Their conviction in the approach was born out of compatibility with their values. The common basis of these value systems seemed to relate to human flourishing and human rights although few directly referred to formalised human rights. For example, they felt that young people had the right to influence services that affected them and/or that they were entitled to sexual wellbeing, which shouldn’t be a taboo subject. They described their commitment to positive services in emotive terms - they had a ‘passion’ to make things ‘better’. Such emotional attachment, where present, served to reinforce their belief in positive approaches and subsequently their commitment to taking action.

**Fig. 3:**
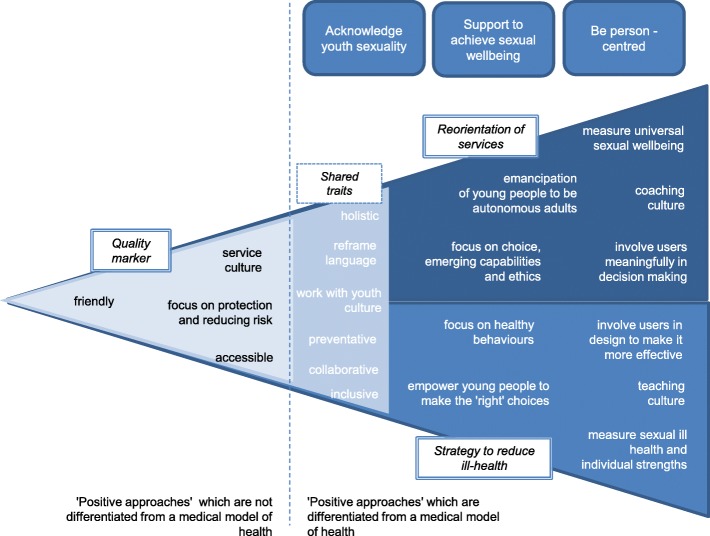
Interpretations of positive services

These three interpretations are illustrated in Fig. 3.

This illustration is a simplification of reality; the picture is muddied because:

- Some aspects of positive approaches are more compatible with people’s core values than others; for example, most were confident talking about building relationship skills, but some felt discussion of sexual pleasure distinctly challenging.
- Individuals’ objectives are multiple and sometimes incommensurable, for example between protecting young people from their vulnerability versus supporting young people to make their own choices.
- Some do act in line with principles and characteristics of a positive approach, despite not having much conviction in it.

#### Refined theories relating to clarity and conviction

NPT [51] posits that *‘differentiation*’ is one process necessary for embedding new practices in an organisation. The data, however, indicated two processes of gaining clarity between positive and other models of care and that interpretations are contingent on prior experience and exposure to ideas. Theories of Transformative [65] and Experiential [66] Learning were therefore incorporated to support an understanding of underlying causal processes.

1. A strategy to reduce ill-health

**Fig. 4:**
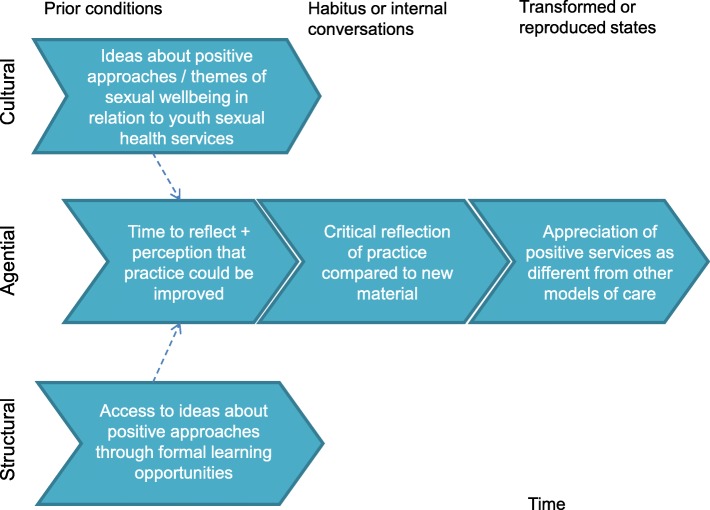
A strategy to reduce ill-health: clarity brought about through transformative learning

The transformative learning configuration relates to availability and access to new material from an external source, real and perceived time for reflection and a perception that practice could be improved (Fig. 4). These collectively trigger critical reflection on practice and an appreciation of positive services as different from other models [65]. The empirical findings suggest that this form of learning was often observed in those in more senior decision-making roles, such as commissioners or senior managers.

**Fig. 5:**
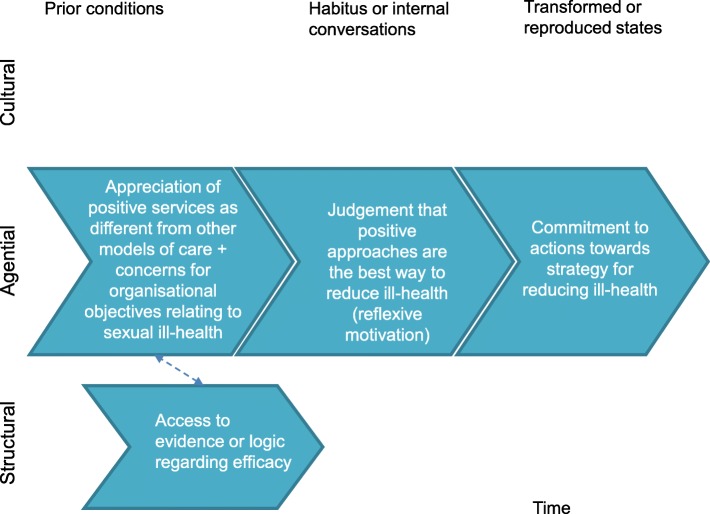
A strategy to reduce ill health: conviction brought about through synergy with objectives

Where decision makers have concern for organisational objectives (which in this case concern reductions in sexual ill-health) a positive approach may be explicitly judged against evidence or logic for its synergy with those objectives. A positive judgement may drive conviction in and commitment to the approach. *Conviction* here can be related to the concept of *‘enrolment’* from NPT [67] and *‘reflexive’* motivation from the COM-B model of behaviour change [68] (Fig. 5).

2) A means to promote choice and capabilities

**Fig. 6:**
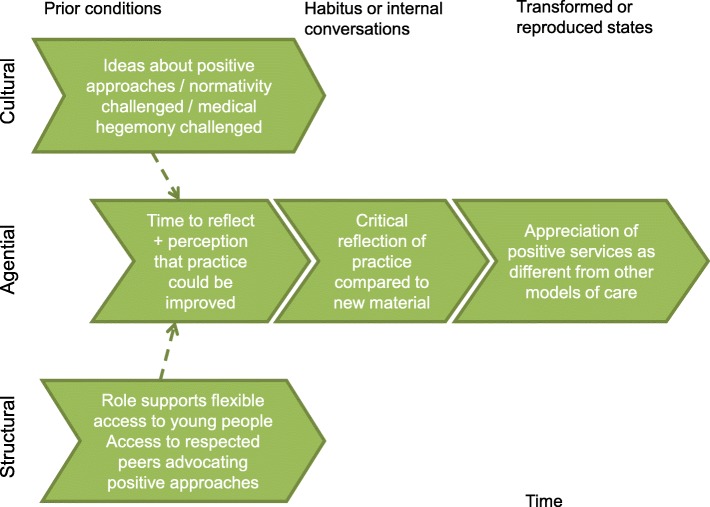
A means to promote choice and capabilities: clarity brought about through experiential learning

The experiential learning [66] configuration relates to working with young people to support their wellbeing, mostly in settings outside clinical practice where practitioners had felt they had greater time and flexibility. It also reflects informal learning opportunities such as discussion with respected peers or personal confrontation with heteronormativity in situations outside professional roles [66]. It should be noted, that the same mechanism, operating at an individual level, is proposed to bring about clarity, that is, critical reflection on practice leading to an appreciation of positive services as different from other models (Fig. 6).

**Fig. 7:**
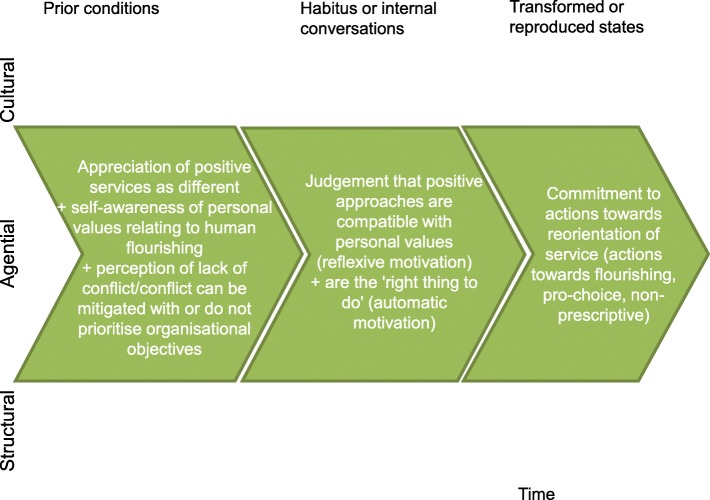
A means to promote choice and capabilities: conviction brought about through compatibility with values

The data highlighted that staff’s conviction in positive approaches as a *means to promote choice and capabilities* is born out of *compatibility with values* concerned with one or more aspects of ‘human flourishing’. In this sense it is related to the concept of ‘*automatic motivation’* from COM-B where pursuing a positive approach feels like the right thing to do. They also possess a belief that they can play a role in supporting young people to achieve sexual wellbeing. This was contingent on whether they believe there is no perceived conflict with organisational objectives, or that such conflicts can be mitigated, or that they do not prioritise the organisational objectives (Fig. 7).

The possible actions and subsequent impact individuals could have differed according to their role and relative power within the system; we turn to this next.

### Coherence

The previous section discussed the first two theory themes, clarity and conviction. This section discusses the theory theme of coherence. Theories relating to coherence were originally developed using the conceptual model, with particular reference to the Morphogenetic Approach [45] and NPT [51].

#### Empirical evidence relating to cultural coherence

Local buy-in was supported when key principles of positive approaches, were shared amongst social actors, particularly by those who had some influence in the system. This seemed to be the case whether conviction was in either the *strategy* or *means to promote capabilities* interpretation - as long as there was agreement.

In *‘Stadford’*, the core team spent time together learning about positive approaches, for example, by visiting the Netherlands and via a community-wide, multi-disciplinary conference. Those who held similar interpretations and convictions formed collectives.

These processes triggered perceptions that others agreed with their beliefs which in turn generated a faith that others would act with them. This appeared to be a stimulus for action.

*"I think that if you are passionate about what you are doing, which I am, and you are clear that you are here to improve the situation and well-being for the whole population, not to please people, and you feel you have the support to do that…and we have that here at every level of the local authority" (Commissioner, 'Stadford').*

Conversely, when the community and clinical services were integrated in *‘Ponston’*, the community practitioners felt they had been ‘taken over’ by clinical services. There was no sense of shared understanding in or commitment to positive approaches. Positive approaches were not pursued because, despite there being a number of individuals who held a conviction within the organisation, their views were diluted. In this case, an interpretation based on values and experience as opposed to ‘evidence’ was particularly precarious because alternate ideas, for example to prioritise medical capability in services, could be presented with equal conviction alongside an evidence base.

#### Empirical evidence relating to structural coherence

Three areas for collective action and contextual integration will be described next in terms of their compatibility or otherwise with the interpretations. These are: evidence and accountability, users’ participation in design and evaluation and integrated provision.

- 1) Evidence and accountability

In all locations, decision makers highlighted the necessity of measuring the impact of any new approach to justify its continued funding. In these six cases ‘impact’ was primarily measured against national public health indicators relating to a narrow set of sexual health outcomes. In *‘Stadford’* and *‘Rissfield’,* for example, tension was observed between this requirement and the ability to measure the effectiveness of positive approaches which by their nature are aimed at addressing broader factors such as cultural stigma, discriminatory processes, developing young people’s skills, knowledge and self-esteem. These may be related in complex and non-predictable ways to health outcomes but causation as well as confounding factors are challenging to measure experimentally.

*"how on earth do you measure someone changing their behaviour as a result of some of the information you have given them?" (Commissioner, 'Stadford').*

In contrast, medical and individualised approaches may have a more reliable demonstrable effect, for example, there is evidence that Long Acting Reversible Contraception reduces the under-18 conception rate and these medical approaches may be prioritised by services held accountable to this indicator score.

Additionally, no validated measurements for young people’s sexual wellbeing, which may pertain to a broader suite of concepts, were available, and tools were considered difficult to develop given the highly individual nature of such a state. The implementation teams who recognised the limitations of existing accountability frameworks, were exploring different ways to evidence their activities, but within this study no substitute to health outcomes was identified. The challenges of evidencing the impact of positive activities on health outcomes (which were required of the commissioned services) appeared in some cases to dilute the local level buy-in to positive approaches, particularly for individuals who were held accountable to them.

- 2) User participation in design and evaluation

In Oregon, *‘Stadford’* and London the involvement of users was a cornerstone for implementing positive services. There were two primary reasons for this. Where the primary interpretation was for positive services to be a *strategy to reduce sexual ill-health* young people’s participation was to ensure that services were effective and designed to be culturally relevant.

*"without young people's input they risked spending all that money and ending up with something no young person would use!" (Local Authority Advisor, 'Stadford').*

Where the primary interpretation was for positive services to be a *means to promoting choice and capabilities*, young people’s participation was seen additionally as a moral imperative, that services for them should be influenced by and accountable to them.

The latter application, however, posed difficulties for decision makers, for example in *‘Rissfield’* in reaching the right young people…

*"The youth council consists of a very specific type of young people…it would be the difficulties around getting a cross section of representation."*

…second, how to allow them to influence meaningfully,

*"[it's also about] the mind-set of us, as commissioners, relinquishing a bit of control…"*

…third, regarding the issue of governance,

*"… if it did fail, how would you ensure the safety of the service users?"*

…finally, the perception of risk involved with adopting users’ input

*"…in this kind of climate of funding cuts… I just don't think we have got the luxury of…'have a go and see how it works out' ". (Commissioner, 'Rissfield')*

As a result, whilst the principle of user involvement was recognised, the operationalisation of such activities was more challenging.

- 3) Integrated provision

Collaboration between a range of professionals with broader skills sets was seen as essential for either the *strategy to reduce sexual ill-health* or *means to promote choice and capabilities* interpretations. However, this questioned traditional medical hierarchy and authority. In *‘Rissfield’* this was overcome as the youth service was a nurse-led (as opposed to consultant-led) multi-disciplinary team built around the holistic needs of young people. However, in other locations, particularly those integrated in GUM settings, an increased role for nurses and non-clinical professionals required senior medical staff to cede control and professional authority in addressing the user’s needs. For some, this challenged their sense of purpose and professional identity. For example, MacFarlane et al. [69] reporting on the London case explained that a consultant in London who advocated for the role of community support workers was considered to be ‘betraying’ other consultants who were competing for the same resources. In *‘Ponston’*, integration, of a community service with a traditional clinical service, in the context of reduced funding, resulted in the elimination of the characteristics supporting young people to build skills and positive relationships due to medical treatment priorities.

#### Refined theories relating to coherence

**Fig. 8:**
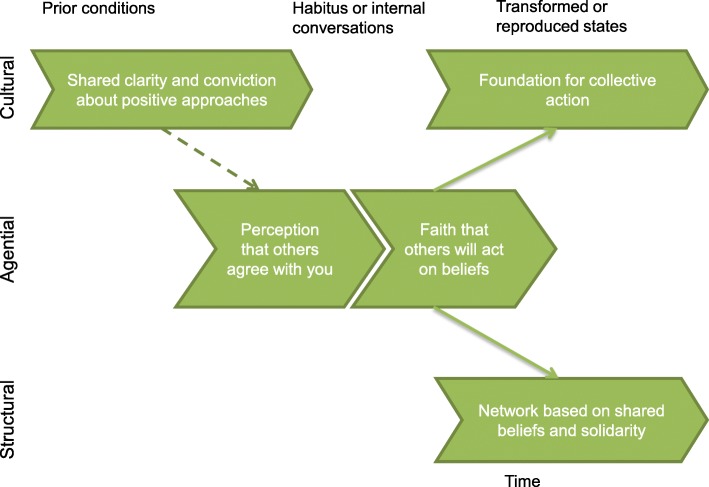
Cultural coherence based on perception of shared understanding and conviction

NPT [51] supports the data in endorsing that shared understanding can lead to collective action. It strengthens one’s own conviction if others agree with you and increases likelihood to act due to expectation that others will support you. This provides a cultural and structural bedrock for action (Fig. 8).

Data suggests shared understanding must be complemented by structural coherence, that is, integration with the specific organisation and government level policy, practices and processes to secure and sustain local buy-in.

**Fig. 9:**
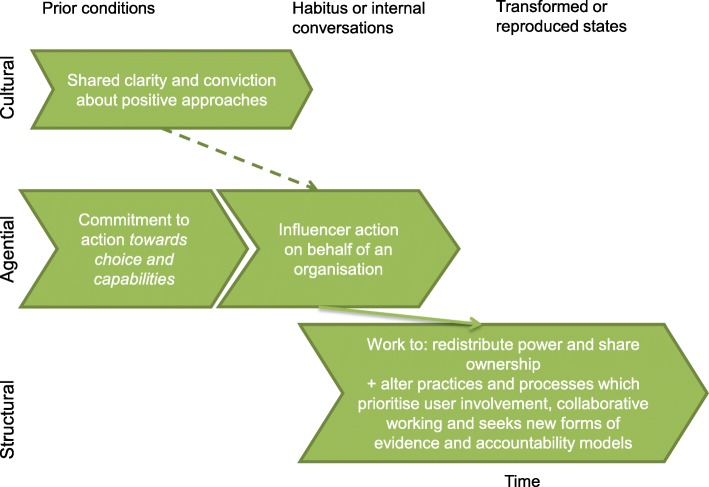
Structural coherence based on ‘means to promote choice and capabilities’

The first of the refined theories relates to positive services as a *means to promote choice and capabilities*. This involves work within different levels of the system to redistribute power and share ownership of decisions with frontline practitioners and young people themselves. Whilst this theory may be logically sound, these structural and cultural barriers mean that an enormous effort would be required to sustain a positive service such as this (Fig. 9).

**Fig. 10:**
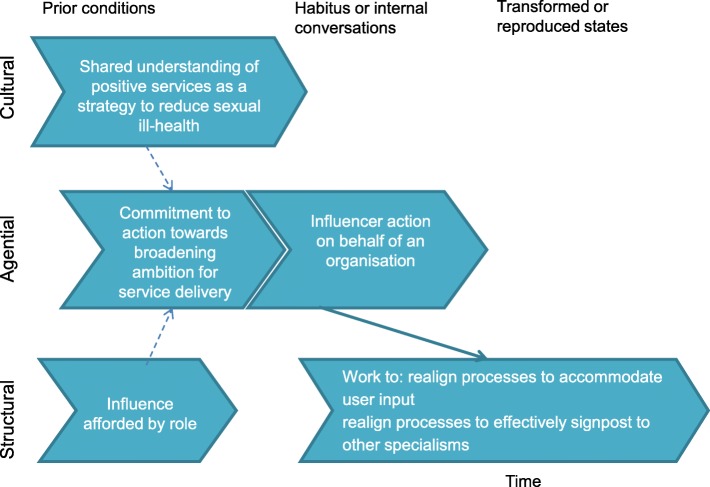
Structural coherence based on ‘strategy to reduce ill health’

Where positive approaches reflect a *strategy to reduce sexual ill-health* participants were able to assimilate positive principles and characteristics more easily within the existing structure. Tensions were alleviated, at least initially, through compromise. Positive approaches were positioned as a way to bring about the outcomes which were predetermined as important by society. User-involvement was positioned as a mechanism for improving the effectiveness of the services; integrated provision was described as a way of moving users around the system to retain a focus on specialisms (Fig. 10).

This elaboration of youth sexual health services was precarious because of the lack of good local evidence on the impact of broader approaches and the threat even this approach poses to existing medical hierarchies.

## Discussion

This article concerns local buy-in to positive youth sexual health service delivery detailing one aspect of what would be a larger implementation chain.

In summary, the following partial, fallible theory suggests that:

- Where individuals have access to ideas about positive services and are able to critically reflect on these ideas compared to current practice they will have *clarity* about what positive services mean and how this might affect their day to day role.
- Where they perceive that this approach is either compatible with their values and/or provide a means to reach their objectives they will have *conviction* in positive approaches.
- Where this conviction is shared with other individuals, there is *cultural coherence.* This provides a platform on which changes towards positive services can be based.
- Where work is compatible with existing processes and practices, s*tructural coherence* supports changes to processes and practices or work is done to change existing processes and practices.

This study highlights that contextual variation concerned with; job roles, professional background, career experience and freedom, time and opportunity to explore different ideas, and access to training, affect what learning can occur. In addition, exposure to background theories about young people’s sexual health affect how new ideas are interpreted [70]. This variation is arguably compounded by the lack of a unified view of what positive approaches are in academic or policy sources. Williams and colleagues maintain that this diversity is a strength because it allows for local interpretation [23]. However, this study has demonstrated that variation in ideas at a local level impedes implementation. Cultural coherence, that is, coming to a shared view, is, arguably, essential. In addition, bringing to the surface deeply held beliefs about sexuality in relation to young people may also help develop an approach which is consistent with values or highlight personal values which may need to be addressed in order to avoid cognitive dissonance and resulting inaction.

In this study, various threats from the local and national structures exacerbated difficulties in gaining local buy-in or sustaining the approach where it is tentatively established. This is arguably compounded in the concurrent English context of austerity measures where services within the study prioritised the clinical provision (although these too were asked to make efficiencies). This can be explained by socio-cultural interaction beyond the services themselves, such as the political climate and medical hegemony; these may dictate the need for narrow outcome measures (associated with outcomes which reflect poor medical and socio-economic status and hence a drain on the economy), accountability (which drives bureaucratic processes, and subsequent lack of freedom to pursue new ideas) and may restrict collaborative working on the basis that it might undermine professional status.

Structural elaboration, as opposed to transformation, may take place if there is compatibility with the dominant models of sexual health in practice. This perceived compatibility may represent a compromise, between the core principles of positive approaches and medical models [45]. The case studies highlight that this position is highly fragile and liable to retrench to a solely medical model if key personnel move on, or if broadened activities are threatened by funding cuts. This suggests that positive services may only become a reality if work is done simultaneously at every level of the system to appreciate a broader notion of human flourishing and facilitate the building of young people’s capabilities as opposed to focussing on a narrow range of health outcomes. These results support calls to broaden the measurements and accountability frameworks by which success is measured [13] and ensure the active participation and influence of young people in design and evaluation of services [71].

This study’s strengths lie in its attention to decision making at different levels of a service. This highlighted tensions which restricted the advocates’ ambitions to transform due to pervasive structural and cultural mechanisms. This was arguably, facilitated by the novel explanatory framework employed to describe the theories which expanded the search for mechanisms beyond the reasoning of individuals in response to a programmes resources. The applicability of this explanatory framework for articulating realist theories to other projects warrants further exploration.

The study’s limitations relate primarily to the small number of cases considered. The lack of cases reported in the literature, resulted in a limited sample. Non-uniformity in the cases studied means the theories developed should be considered as tentative. Our decision to return to the same cases to test emerging theories should, however, serve to increase their credibility. The study primarily used data from the UK when services were facing financial pressures due to austerity measures imposed by parliament. This may limit the applicability of some aspects of the theory to other nations; however, we feel that our attention to developing middle range theory supports the transferability of findings to other contexts.

## Conclusions

Dissonance between individual level ambition and established cultural norms and structural processes linked to accountability frameworks and medical hegemony may restrict the successful implementation of positive youth sexual health services. This may help to explain the current limited progress highlighted by WHO [1]. Future initiatives should be theoretically informed and underpinned by a clear values set to address incongruence at societal, organisational and interpersonal levels to stimulate change.

## Additional files

Additional file 1: Search strategy. Search statement, strategy, details of inclusion, exclusion criteria and modified Prisma diagram. (DOCX 66 kb)

Additional file 2: Early themes and Initial Rough Programme Theories. Initial theories that were derived from the literature based case studies and middle range theories. (DOCX 22 kb)

Additional file 3: Study Sources. This file includes the data sources from the academic literature, grey literature and interview and workshop participants. (DOCX 59 kb)

## Data Availability

The datasets generated and analysed during the current study are not publicly available due risk to individual privacy but pseudonymised data sets are available from the corresponding author on reasonable request. This article contains some textual overlap with the first author’s thesis which is available on a non-exclusive license.

## Abbreviations

CMOc: Context mechanism outcome configuration
COM-B: Capability opportunity motivation = behaviour (change)
GUM: Genito-urinary medicine
IPRT: Initial rough programme theories
MRT: Middle range theory
NPT: Normalisation Process Theory
Positive services: Positive, comprehensive youth sexual health services
RPT: Refined programme theories

## Footnotes

1 Theory development began in 2015 utilising the iteration of NPT illustrated in the 2009, 2011 and to a lesser extent 2013, papers.
