# Supplementary material for: Clarity, conviction and coherence supports buy-in to positive youth sexual health services: focused results from a realist evaluation
Source: BMC Health Serv Res. 2019 Jul 19;19:503. doi: 10.1186/s12913-019-4298-4 (PMC6642563; doi:10.1186/s12913-019-4298-4)
Supplement: Supplementary file 1 — Search strategy. Search statement, strategy, details of inclusion, exclusion criteria and modified Prisma diagram. (DOCX 66 kb) [file 12913_2019_4298_MOESM1_ESM.docx]

**Additional File 1: Search Strategy**

### 1. Academic literature search statement

The aim of the search was to identify case studies in the academic literature to describe how youth sexual health services characterise a positive approach in practice and extract theory propositions about how these are operationalised. The search of the academic databases therefore aimed to identify accounts of youth sexual health services which explicitly claim a positive approach to youth sexual health sexuality.

### 2. Academic literature search strategy

A broad search strategy was developed to capture literature about positive youth sexual health services from a wide range of sources. This was an iterative process which fitted the exploratory nature of the review question. The key concepts were derived from the search statement initially. The first search terms were thus established as: "sexual health", "sex positive", "young people" and "service". Other synonyms and applicable terms were added including "holistic" or "rights" which may imply comparable approaches to "positive".

The index of MEDLINE was also used to identify further potential Medical Subject Headings for "sexual health". Finally, this search strategy was piloted on one academic database, Scopus. A foray into the literature identified through this initial search allowed the expansion of the search terms to include controlled vocabulary; "adolescence", and applicable terms; "integrated", "preventative" and "comprehensive". The search was built by combining terms utilising the connector words AND between key concepts and OR between synonyms. The final search terms are illustrated in Table 1.

The strategy involved searching electronic databases; citation and reference searches; and contact with authors. This breadth was necessary because accounts of local service approaches or transformations, which may be messy, organic and multi-faceted, may rarely appear in detail in many academic journals, which favour experimental designs and have restrictive word limits.

###### Table 1: Search terms for positive youth sexual health services

|  | Key Concept:  "Sexual Health" | Key Concept:  "Sex Positive" | Key Concept: "Young people" | Key Concept:  Service* |
| --- | --- | --- | --- | --- |
| Synonyms  Use 'OR' | "Reproductive Health" | "Sex-positive" | Young | Intervention* |
|  |  | "Sexual subjectivit*" | Youth | Framework* |
|  |  | "Sexual rights" | Teen* | Paradigm* |
|  |  | Holistic | Adolescen* | Program* |
|  |  | Integrated |  | Model* |
|  |  | "Human rights" |  | Trial* |
|  |  | "Reproductive rights" |  | Pilot* |
|  |  | Preventative |  |  |
|  |  | Comprehensive |  |  |

### 3. Academic literature searches

#### Database searches

The search was run on four academic databases: MEDLINE, CINAHL Complete, Scopus and PsychINFO on 21.04.2015.

#### Inclusion / exclusion criteria

The search identified 1162 articles after removing duplicates. The following inclusion and exclusion criteria were then applied to select the most relevant sources.

*Contemporaneousness:*  A start date was not included for three reasons. First because the relative modernity of the concepts of sexual health and sex-positivity would provide a natural start towards the latter half of the 20th century. Second, it might have been possible to unwittingly discount positive approaches, established a number of years ago, when academic accounts might have been generated, that were still operating. Third, as services are influenced by historical, political and societal norms and values, progression towards certain ideas is not necessarily chronologically linear, such that services may have been more positive in the past compared with now.

*Geography:* Only accounts from developed countries were included. This was primarily because of the need to limit the possible confounding influences on service design and delivery which may be critical in developing countries but less so in the developed world. For example, decision making about service design and delivery in the developing world may be more concerned with transport infrastructure, vast inequalities and the need to meet other basic needs such as accommodation and food. These issues could still be present in many developed countries but are arguably less acutely influential.

*Study design:* As noted above, there were no exclusions on the basis of article type or study design. Accounts of service design and delivery could have included a range of sources including opinion pieces, primary studies or evidence summaries.

*Service characteristics:* Only articles which related to routine sexual healthcare or support were included. As such there were four specific exclusions. These were articles which concerned ***only***:

- Education or information. This, although important, was outside the scope of the study.
- Specific aspects of sexual health, such as under-18 conceptions or HIV. This was to ensure relevance of the study to a broad conceptualisation of sexual health and how this had been applied, if at all, to practice.
- Youth development programmes. This is a generic term used to refer to formal programmes or interventions often lasting several sessions to which young people are referred to or sign up for. The aims and intentions of these programmes are often to affect specific behaviour change, for specific clients, which was also outside the scope of the study.
- One-off interventions, which would not support understanding of the characteristics of universal services.

Youth sexual health services were included which distinguished their approach from other models of care with reference to positive sexual-wellbeing and related approaches labelled 'holistic' and 'comprehensive'.

*Clients:*  Articles relating to services that provided universal services for young people, under the age of twenty five, in either standalone settings or as part of their general provision were included. Articles which described services targeted at specific client populations *only*, such as care leavers or young people identifying as LGBT were excluded.

After applying the inclusion and exclusion criteria, two articles in the academic literature referred to positive youth sexual health services.

Later, in reviewing the realist methods papers, a further article was found through serendipity. This had not been picked up in the initial search strategy because it related to whole system transformation of three services and not just youth sexual health. Neither sexual health, nor young people, or synonyms were mentioned in the title, abstract or key words. On reading the detailed report it was clear that transformation to a positive approach for all clients, including young people, in the general provision, was the intention of the initiative, thus it met the inclusion criteria.

An alert on academic databases used to conduct the original search was set up to provide notification for any further articles that might come to light over the remainder of the research period. This did not highlight any other positive youth sexual health services.

#### Reference and citation searches

The three articles were about three unique services which are referred to as Oregon, Lothian and London case studies. Further reference and citation searches for kin papers, in order to gain contextual richness for each of these case studies were conducted. This located multiple accounts of the same service transformation. The kin papers included internal reports, evaluation reports, promotional material and other academic papers. They related to different time periods. This allowed us to compare and contrast descriptions of the services from different perspectives (and for different purposes). This was useful for developing a deeper understanding of the characteristics reported and highlighted any inconsistencies.

In addition, contact with authors was possible for two of the case studies. For Oregon, an email exchange between KS and a current employee of the local public health department produced further detail regarding the development and characteristics of that service and directed us to other resources that they had used to demonstrate impact. For London, an email request was made to one of the authors who directed me to a number of further papers relevant to my theory development. The full search process is illustrated in the modified Prisma diagram in Figure 1.


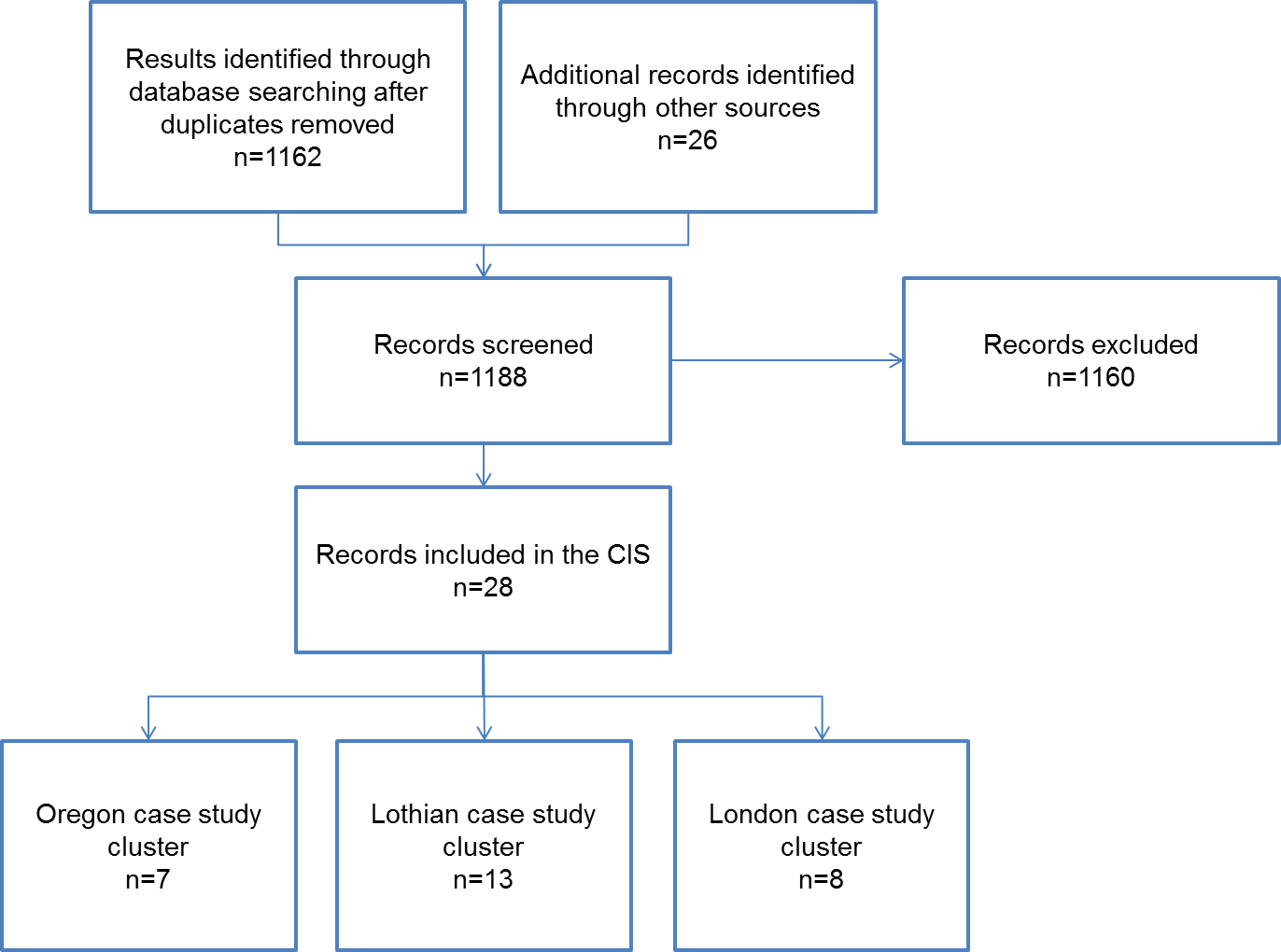


##### Figure 1 Modified PRISMA diagram illustrating the search results
