# Supplementary material for: Clarity, conviction and coherence supports buy-in to positive youth sexual health services: focused results from a realist evaluation
Source: BMC Health Serv Res. 2019 Jul 19;19:503. doi: 10.1186/s12913-019-4298-4 (PMC6642563; doi:10.1186/s12913-019-4298-4)
Supplement: Supplementary file 2 — Early themes and Initial Rough Programme Theories. Initial theories that were derived from the literature based case studies and middle range theories. (DOCX 22 kb) [file 12913_2019_4298_MOESM2_ESM.docx]

**Early Theory Themes and Initial Rough Programme Theories**

Early theory themes derived from initial literature reviews and conceptual framework of theories: Morphogenetic Approach (MA), Normalisation Process Theory (NPT) and COM-B from the Behaviour Change Wheel.

| Conviction | | | | |
| --- | --- | --- | --- | --- |
| Proposition | Data | MA | NPT | COM-B |
| When commissioners, practitioners and managers understand what is meant by positive approaches (P.A.). | * |  | * |  |
| and recognise a need to improve local young people's sexual health | * |  |  | * |
| and see P.A. as a means to improve young people's sexual health | * | * | * | * |
| they will develop conviction that it is most likely to work | * |  | * | * |
| and explore ways of implementing the approach | * |  | * |  |

| Consistent with existing policy | | | | |
| --- | --- | --- | --- | --- |
| Proposition | Data | MA | NPT | COM-B |
| Where pre-existing policy mandates regarding youth sexual health reflect risk-reduction discourses | * |  |  |  |
| and there is a need for accountability | * |  |  |  |
| commissioners, practitioners and managers who intend to embed a positive approach in their work | * | * | * | * |
| will strive to find coherence between convictions, actions and short-term self-preservation goals | * | * |  |  |

| Shared values | | | | |
| --- | --- | --- | --- | --- |
| Proposition | Data | MA | NPT | COM-B |
| When commissioners, practitioners and managers who intend to embed a positive approach in their work | * | * | * | * |
| share an understanding of positive principles and characteristics with other local decision makers | * | * | * |  |
| and pursue the same goals | * | * | * |  |
| they will be motivated to work together as this will enhance their chances of success |  | * |  | * |

| Integration with other contextual features | | | | |
| --- | --- | --- | --- | --- |
| Proposition | Data | MA | NPT | COM-B |
| When commissioners, practitioners and managers who intend to embed a positive approach in their work | * | * | * | * |
| they will develop local policy, practices and processes which makes explicit overarching positive aims and ambitions, roles and responsibilities | * | * | * |  |
| thereby increasing coherence surrounding positive approaches and individuals roles within it | * | * | * | * |

| Evidence-based practice | | | | |
| --- | --- | --- | --- | --- |
| Proposition | Data | MA | NPT | COM-B |
| When commissioners, practitioners and managers who intend to embed a positive approach in their work | * | * | * | * |
| and there is a need for accountability | * |  | * |  |
| they will seek to develop a broad range of data to justify efficacy and impact of positive approaches | * |  |  |  |
| in order that it might continue to be supported | * | * | * |  |

| Devolved decision making | | | | |
| --- | --- | --- | --- | --- |
| Proposition | Data | MA | NPT | COM-B |
| When practitioners who intend to embed a positive approach in their work | * | * | * | * |
| have autonomy to exercise discretion | * |  |  |  |
| and feel capable, motivated to use positive approaches | * |  | * | * |
| will be able to utilise positive approaches where appropriate | * |  |  | * |

| Young people's voice at the centre of decision-making | | | | |
| --- | --- | --- | --- | --- |
| Proposition | Data | MA | NPT | COM-B |
| When commissioners, practitioners and managers who intend to embed a positive approach in their work | * | * | * | * |
| they will recognise that they can't hold all the answers to meet the needs of young people | * | * |  |  |
| and will engage young people in service design and decision making | * | * |  |  |

| Tension between practice requirements | | | | |
| --- | --- | --- | --- | --- |
| Proposition | Data | MA | NPT | COM-B |
| When commissioners, practitioners and managers who intend to embed a positive approach in their work | * | * | * | * |
| recognise a potential tension between this and other aspects of their work such as safeguarding | * | * | * |  |
| they will explore ways of reducing the tension | * | * | * |  |
| or one aspect will be side-lined for another | * | * | * |  |

**Initial Rough Programme Theories**

Initial rough programme theories developed following a prioritisation exercise which demonstrated ‘buy-in’ as a critical step in implementation of positive services.

IRPT 1a: Differentiation

Where principles and characteristics of positive approaches are made explicit, via training or illustrations of other models of care, commissioners, managers and practitioners will be able to distinguish this approach from other models of care.

IRPT1b: Individual specification

When directed to do so, commissioners, managers and practitioners will be able to make sense of the principles and characteristics of a positive approach and understand how it might affect their day to day roles and responsibilities

IRPT 2a: Synergy with objectives

When practitioners engage with a positive approach, they will judge the extent to which it will help them meet their ends. These may be personal or organisational objectives. They will only have the intention to embed its principles and features in their own work if they believe it meets their ends or those of others.

IRPT 2b: Compatibility with values

When practitioners engage with a positive approach, they will begin to judge it against their values and only have the intention to embed its principles and characteristics in their own work if they deem the values on which it is based to be compatible.

IRPT2c: Conviction based on synergy with objectives and compatibility with values

When practitioners engage with a positive approach and judge it as an appropriate way to meet their objectives and as compatible with their values they will develop a strongly held conviction in its promise. This will strengthen the intention to embed the principles and features in their work.

IRPT 3a: Cultural coherence a)

When commissioners, managers and practitioners, who intend to embed a positive approach in their work share an understanding with other local decision makers and pursue the same goals, they will be motivated to work together as this will enhance their chances of success

IRPT 3b: Cultural coherence b)

When there is a clash between the existing cultural and the new idea and the advocacy group have high corporate agency, by virtue of their own role and status, ideas and processes relating to the more traditional approach may be 'eliminated' and a positive approach imposed.

IRPT 3c: Evidence and accountability a)

Where evidence-based practice is strongly recommended, positive approaches may not be deemed acceptable, as they may not have a suitably strong evidence base. In these cases, practitioners are compelled to develop a credible, alternative or additional evidence base to substantiate its wider claims.

IRPT 3d: Evidence and accountability b)

When commissioners, managers and practitioners believe that a positive approach is the best way to meet the pre-existing organisational objectives such as reducing teenage conceptions and STI transmission rates (theory 2b), work will be orientated towards and evaluated against measures related to these outcomes. If outcomes do not improve, this may pose a threat to a positive approach because organisations are orientated towards demonstrable successes.

IRPT 3e: Integrated provision

When all individuals, with a role in the delivery of youth sexual health services, have a shared, broad understanding and conviction in positive services (theory 3a and b), they will intend to work to integrate provision with other stakeholders in the network.

Theory 3f: User led design and evaluation

When all individuals, with a role in the delivery of youth sexual health services, have a shared, broad understanding and conviction in positive services, they will work to include users in the design and evaluation of their service because: it is right to consult people on services that affect them, and/or this may provide insight to improve service design and/or this may provide evidence to support the service transformation.

If achieved, this can prove to be a catalyst for change, improving the practical aspects of the approach and strengthening resolve for it, particularly where practitioners are concerned with promoting the rights of young people (theory 2b).
