# Supplementary material for: Clarity, conviction and coherence supports buy-in to positive youth sexual health services: focused results from a realist evaluation
Source: BMC Health Serv Res. 2019 Jul 19;19:503. doi: 10.1186/s12913-019-4298-4 (PMC6642563; doi:10.1186/s12913-019-4298-4)
Supplement: Supplementary file 3 — Study Sources. This file includes the data sources from the academic literature, grey literature and interview and workshop participants. (DOCX 59 kb) [file 12913_2019_4298_MOESM3_ESM.docx]

**Additional File 3: Sources utilised in the realist evaluation of positive youth sexual health services**

**Data sources retrieved from the academic literature search for secondary case studies.**

| Case study | Title (reference) | Year | Provenance | Topic area |
| --- | --- | --- | --- | --- |
| Oregon | Oregon Youth Sexual Health Plan (Oregon Department of Human Services, 2009a) | 2009 | Grey (reference) | State-wide strategy document for youth sexual health |
|  | Oregon Youth Sexual Health Plan appendices (Oregon Department of Human Services, 2009b) | 2009 | Grey (reference) | Appendices including reports of specific data gathering activities to inform the Oregon Youth Sexual Health Plan |
|  | Shifting the paradigm in Oregon from teen pregnancy prevention to youth sexual health (Nystrom, Duke, & Victor, 2013) | 2013 | Academic database (search strategy) | Public health report on the Oregon Youth Sexual Health Plan. |
|  | Youth participatory action research curriculum (Institute for Community Research, 2014) | 2014 | Grey (hand searching) | Adapted curriculum report to support practitioners undertake youth participatory action research to promoted positive youth development in Oregon |
|  | Oregon Youth Sexual Health Plan: Five Year Update (Oregon Public Health Division, 2014) | 2014 | Grey (hand searching | Progress report on Oregon Youth Sexual Health Plan |
|  | Personal communication by email (anonymous) | 2015 | Email exchange | Detail by email given in response to specific questions posed by the researcher |
|  | Oregon Guidance for the Provision of High-Quality Contraception Services: a Clinic Self-Assessment Tool (Oregon Preventative Reproductiv Health Advisory Council, 2017) | 2017 | Grey (hand searching) | Self-assessment and resource pack for sexual health clinics delivering contraceptive services including guidance for young people |
| Lothian | Enhancing Sexual Wellbeing in Scotland: A Sexual Health and Relationships Strategy (Scottish Executive, 2003) | 2003 | Grey (hand searching) | Consultation document on the draft National Sexual Health and Relationships Strategy |
|  | All I want. A review of specialist sexual health services for young people: young people's report (Healthy Respect, 2004) | 2004 | Grey (hand searching) | Report of research carried out to understand young people's views about sexual health clinics and drop-in services |
|  | Respect and Responsibility Strategy and Action Plan for Improving Sexual Health (Scottish Executive, 2005) | 2005 | Grey (hand searching) | Scottish National Sexual Health Strategy |
|  | Final summary report of the external evaluation of Healthy Respect, a national health demonstration project (Tucker, Penney, Van Teijlingen, Shucksmith, & Philip, 2005) | 2005 | Grey (hand searching) | External evaluation of the Healthy Respect National Demonstration Project. Observation period November 2000-2004 |
|  | Healthy Respect Phase Two (Healthy Respect, 2005) | 2005 | Grey (hand searching) | Proposal for phase two of the Healthy Respect National Demonstration Project on Young People's Sexual Health |
|  | An evaluation of the impact of a national health demonstration project on testing and management for chlamydia trachomatis infections in two regions of Scotland (Penney, Brace, Cameron, & Tucker, 2005) | 2005 | Academic database - (reference searching) | Clinical audit of practice against national standards for good quality care comparing intervention region with another region |
|  | Health demonstration projects: Evaluating a community-based health intervention programme to improve young people’s sexual health (Tucker, Van Teijlingen, Philip, Shucksmith, & Penney, 2006) | 2006 | Academic database (search strategy) | Methodological opinion piece on evaluating the Healthy Respect National Demonstration Project |
|  | Healthy Respect drop-ins: a guide to accredited status and how to achieve it (Healthy Respect, 2007) | 2007 | Grey (hand searching) | Guidance for organisations that want to become Healthy Respect accredited |
|  | The effect of the national demonstration project Healthy Respect on teenage sexual health behaviour (Tucker et al., 2007) | 2007 | Academic database - (citation searching) | Primary research report of the before and after cross-sectional surveys of secondary school pupils in 10 Healthy Respect intervention schools |
|  | Tackling sexual health inequalities: work in progress with young people at risk (Paterson, 2007) | 2007 | Academic database - (citation searching) | Description of a range of pilot initiatives underway and evaluation processes to tackle sexual health inequalities |
|  | Healthy Respect Dissemination Programme (NHS Health Scotland, 2010) | 2010 | Grey (hand searching) | Final report of the programme developed to share implementation, experiences and evaluation reports from the Healthy Respect National Demonstration Project |
|  | Evaluation of healthy respect phase two: final report (Elliott et al., 2010) | 2010 | Grey (hand searching) | Final evaluation report of phase two of the Healthy Respect National Demonstration Project |
|  | Healthy Respect Website (Healthy Respect, 2017) | 2017 | Google search | Website |
| London | A new approach to sexual health in South London (Kings College Hospital Media Team, 2007) | 2007 | Google search | Press release |
|  | King's Sexual Health Centre wins top NHS Award (press release Kings College Hospital Media Team 2007) | 2008 | Google search | Press release |
|  | The Modernisation Initiative: independent evaluation final report (Greenhalgh et al., 2008) | 2008 | Grey (contact with authors) | Final evaluation report of the Modernisation Initiative |
|  | How do you modernize a health service? A realist evaluation of whole-scale transformation in London (Greenhalgh et al., 2009) | 2009 | Academic database - (serendipity) | Realist evaluation of the modernisation initiative |
|  | An independent evaluation of Lambeth's modernised sexual health service delivery model (Miles, 2010) | 2010 | Grey (hand searching) | Evaluation of the sexual health service model that had been implemented as part of the Modernisation Initiative (evaluation period December 2009 - 2010) |
|  | A new workforce in the making? A case study of strategic human resource management in a whole-system change effort in healthcare (Macfarlane et al., 2011) | 2011 | Academic database (citation searching) | Realist evaluation of whole-system workforce development as part of the Modernisation Initiative evaluation |
|  | If we build it, will it stay? A case study of the sustainability of whole-system change in London (Greenhalgh, MacFarlane, Barton-Sweeney, & Woodard, 2012) | 2012 | Academic database (citation searching) | Mixed-method case study of a three year follow up of the Modernisation Initiative |
|  | Achieving and sustaining profound institutional change in healthcare: case study using neo-institutional theory (Macfarlane, Barton-Sweeney, Woodard, & Greenhalgh, 2013) | 2013 | Academic database (citation searching) | Secondary analysis of case study data derived from the Modernisation Initiative evaluation |

**Data sources retrieved from English policy, professional and guidance websites.**

| Category | Title (reference) | Year (Year of operation) | Provenance | Topic area (as defined within the document) |
| --- | --- | --- | --- | --- |
| Current National policy / Acts of Parliament | Sex and Relationship Education Guidance (Department for Education and Employment, 2000) | 2000  (ongoing) | Reference Searching | Guidance for sex and relationship education in schools. Arising out of the Personal, Social and Health Education (PSHE) framework and Teenage Pregnancy Strategy |
|  | Health and Social Care Act 2012  (Department of Health, 2012a) | 2012 (2013) | Google search | Act of Parliament detailing a substantial revision of the NHS. Established Clinical Commissioning Groups, Public Health England and transferred commissioning of sexual health services from the NHS to the local authority |
|  | Framework for Sexual Health Improvement in England (Department of Health, 2013a) | 2013 (ongoing) | Hand searching | Framework document to support those involved in sexual health improvement work together effectively. Replaced the Teenage Pregnancy Strategy and National Strategy for Sexual and Reproductive Health |
|  | Five Year Forward View (NHS England, Care Quality Commission, Health Education England, Monitor, Public Health England, 2014) | 2014  (ongoing) | Hand Searching | Shared vision of national leadership for a better NHS and steps to take to get us there. |
|  | Health promotion for sexual and reproductive health and HIV: strategic action plan, 2016 to 2019 (Public Health England, 2015) | 2015 (2016-2019) | Department of Health website | Strategic action plan setting out Public Health England's approach to improving the public's sexual and reproductive health. Works within the Framework for Sexual Health Improvement in England and the Public Health Outcomes Framework |
|  | Improving young people's health and wellbeing: a framework for public health (Public Health England & Association for Young People’s Health, 2015) | 2015  (ongoing) | Hand Searching | Framework to address the specific needs of young people (10-24 years old) containing practical support to councillors, health and wellbeing boards, commissioners and service providers. |
| Past National policy (selected) | Teenage Pregnancy Strategy (Social Exclusion Unit, 1999) | 1999  (2000-2010) | Reference searching | Report setting out the analysis of the teenage pregnancy in the UK and decisions made to tackle it |
|  | Teenage Pregnancy Strategy: Beyond 2010 (Department for Children Schools and Families, 2011) | 2011 (2011-2013) | Reference searching | Interim strategy document to build on learning of Teenage Pregnancy Strategy |
|  | The National Strategy for Sexual Health and HIV (Department of Health, 2001) | 2001  (2001-2010) | Hand Searching | First national strategy for sexual health and HIV |
|  | Progress and priorities - working together for high quality sexual health (MEDFASH, 2008) | 2008 | Citation searching | Review of the National Strategy for Sexual Health and HIV commissioned by the Independent Advisory Group on Sexual Health and HIV |
| National professional bodies | Better Care, Better Future: a new vision for sexual and reproductive health care in the UK (Faculty of Sexual & Reproductive Healthcare, 2015) | 2015 | Hand searching | Vision statement from the multi-disciplinary membership faculty of sexual and reproductive healthcare professionals |
| National advocacy bodies | Young People (FPA, 2011) | 2011 | Hand searching | Policy statement on young people |
|  | Guidance for using the sexual behaviours traffic light tool (Brook, 2015) | 2015 | Hand searching | Resource to categorise sexual behaviours of young people |
|  | Breaking down the barriers: The need for accountability and integration in sexual health, reproductive health and HIV service in England (All-Party Parliamentary Group on Sexual and Reproductive Health in the UK, 2015) | 2015 | Hand searching | Report of the inquiry to understand the impact of the new structures established as part of the Health and Social Care Act 2012. |
|  | Be Sex : Positive Hello (Brook, 2016) | 2016 | Hand searching | Pack to explain about the Sex:Positive Campaign |
| National Guidance / guidelines | Contraceptive choices for young people (Faculty of Sexual & Reproductive Healthcare, 2010) | 2010  (ongoing) | Reference searching | Guidance for health professionals on providing contraceptive services to young people. Recommendations based on available evidence and expert consensus |
|  | Spotting the signs: a national proforma for identifying risk of child sexual exploitation in sexual health services (Rogstad & Johnston, 2011) | 2011 (ongoing) | Recommend-ation | National proforma to help health professionals working with young people identify and assess the risk of child sexual exploitation (CSE). |
|  | You're Welcome - Quality Criteria for young people friendly health services (Department of Health, 2011) | 2011  (ongoing) | Reference searching | Quality criteria for youth-friendly health services |
|  | Integrated Sexual Health Services: National Service Specification (Department of Health, 2013c) | 2013  (ongoing) | Hand searching | National service specification to help local authorities commission effective, high-quality, integrated sexual health care |
|  | Commissioning Sexual Health services and interventions: best practice for local authorities (Department of Health, 2013b) | 2013  (ongoing) | Hand searching | Guidance document for local authorities to fulfil legal requirements related to sexual health commissioning following the Health and Social Care Act 2012 |
|  | Service standards for sexual and reproductive healthcare (Faculty of Sexual & Reproductive Healthcare Clinical Standards Committee, 2013) | 2013  (ongoing) | Hand searching | Service standards aims at commissioners and providers of services in relation to sexual and reproductive health commissioned of contracted by the NHS |
|  | Making it work: A guide to whole systems commissioning for sexual health, reproductive health and HIV (Public Health England, 2014) | 2014 (ongoing) | Department of Health website | Guide for commissioners of sexual health, reproductive health and HIV services in local government, clinical commissioning groups and NHS England outlining responsibilities set out in the Health and Social Care Act 2012 |
|  | Standards for the management of sexually transmitted infections (STIs) (BASHH & MEDFASH, 2014) | 2014 | Hand searching | Guidance bring together the best practice that people seeking help in relation to STIs are entitled to expect |
|  | Developing strong relationships and positive sexual health (Health, 2014) | 2014  (ongoing) | Citation searching | Pathway and guidance to support school nurses, sexual health service providers and partners working to support the contraceptive and sexual health needs of young people |
|  | Preventing sexually transmitted infections and under-18 conceptions overview (NICE, 2014b) | 2014 (ongoing) | Hand Searching | NICE pathway bringing together all NICE guidance, quality standards and materials to prevent sexually transmitted infections and under 18 conceptions |
|  | Contraceptive services with a focus on young people aged up to 25 overview (NICE, 2014a) | 2014  (ongoing) | Hand Searching | NICE pathway bringing together all NICE guidance, quality standards and materials on contraceptive services for young people aged up to 25 |
|  | Sexual health commissioning in local government (Local Goverment Association & MEDFASH, 2015) | 2015 (ongoing) | Reference searching | Nine cases studies showcasing local government experience of commissioning sexual health services since April 2013 |
|  | Working together to safeguard children. A guide to inter-agency working to safeguard and promote the welfare of children (Department for Education, 2015) | 2015 (ongoing) | Hand Searching | Guidance to help professionals understand what they need to do and what they can expect of one another to safeguard children. |
| Key data and statistics | Public Health Outcomes Framework (Department of Health, 2013d) | 2013 (ongoing) | Hand searching | Overarching vision for public health, outcomes that the NHS, social care, voluntary sector want to achieve and the indicators intended to help to measure progress |
|  | Improving outcomes and supporting transparency (August 2016) | 2016  (ongoing) | Hand searching | Technical specifications of the Public Health Outcomes Framework indicator set. |
|  | Sexually Transmitted Infections and Chlamydia Screening in England, 2016 (Public Health England, 2017) | 2017 (data to 2016, key trends) | Hand searching | Key trends in testing and diagnosis of STIs. |

**Data sources retrieved from the empirical case studies.**

***Sample for cycle two (interviews)***

| Type | 'Ponston' | 'Stadford' | 'Rissfield' |
| --- | --- | --- | --- |
| Past decision makers | 1  (Community Manager) | 1  (Project Manager) | 0 |
| Local authority councillors | 1 | 0 | 1 |
| Local authority commissioners | 1 | 2 | 2 |
| Local authority adviser | 0 | 1 | 0 |
| NHS managers | 2 | 1 | 1 |
| Sexual Health Consultant | 1 | 1 | 1 |
| Nurse | 1 | 2 | 1 |
| Outreach / health promotion officers | 1 | 1 | 1 |
| TOTAL | 8 | 9 | 7^[[1]](#footnote-1)^ |

***Supporting documentation for case studies in cycle 2.***

| Type | 'Ponston' | 'Stadford' | 'Rissfield' |
| --- | --- | --- | --- |
| Grey literature published by agencies responsible for commissioning and delivering sexual health services for young people | - Needs assessment - Annual reporting template - Consultation documents on service restructuring - Local evaluation report | - Board of Directors Report - Integrated Performance Board Report - Children and Young People Annual Report - Invitation to Tender Documents | - Teenage Pregnancy Update - Councillor recommendations for signposting for Young People/Partnership Working - Consultation report on young persons' service redesign - Service specification - Sexual Health Partnership meeting minutes - Terms of Reference: Sexual Health Partnership group - Terms of Reference: RSE group |
| Print media derived from Nexis search | - Local newspaper articles | - Local newspaper articles - National news articles | - Local newspaper articles |
| Social media derived from google search |  | - Blog authored by the local authority advisor x 5 entries |  |
| Academic outputs |  | - Published conference proceedings x 1 - Journal article x 1 - Recorded conference presentation on you tube x 1 |  |
| Trend data | - Office for National Statistics Teenage Conceptions data - PHOF indicators and wider determinants of health from Public Health England | | |
| Field notes | - x 4 meetings |  | - x 2 meetings |

***Sample for cycle three (workshops)***

Participants for each of the case studies

| Case study | 'Ponston' | 'Stadford' | 'Rissfield' |
| --- | --- | --- | --- |
| Number of participants | x 48  inc: sexual health consultants, managers, administrators, nurses and health promotion specialists | x 6  inc: senior sexual health nurses and managers | x 9  inc: youth keyworkers, health promotion specialists, sexual health nurses and managers |

Of which all participated in discussions and 47, across the case studies, returned feedback booklets.

1. The smaller number of interviews achieved in Rissfield reflects the smaller size of this service. [↑](#footnote-ref-1)
